# Editorial: Understanding the impact of AI and technology on the assessment, monitoring, and treatment of neurodevelopmental disorders

**DOI:** 10.3389/fpsyg.2023.1321544

**Published:** 2023-11-01

**Authors:** Daniela Pacella, Belén López-Pérez, Davide Marocco

**Affiliations:** ^1^Department of Public Health, University of Naples Federico II, Naples, Italy; ^2^School of Health Sciences, University of Manchester, Manchester, United Kingdom; ^3^Department of Humanistic Studies, University of Naples Federico II, Naples, Italy

**Keywords:** neurodevelopmental disorders, artificial intelligence, telehealth, assistive technologies, mental health

Neurodevelopmental disorders (NDD) are a group of conditions that originate in the early stages of life but often persist through adulthood. Among them, the most prevalent in Europe and the USA are autism spectrum disorder (ASD), attention-deficit/hyperactivity disorder (ADHD), learning disabilities, and other impairments affecting language and communication. Diagnosing, treating, and monitoring such conditions are usually a complex and costly process especially since these often evolve into chronic conditions. Difficulty and delay in diagnosis and intervention due to the recent COVID-19 pandemic, lack of access to expert therapists and medical staff, or high-cost burden all contribute to poorer therapeutic conditions and outcomes.

In response to the pandemic, there has been a notable surge of telehealth and assistive technology and delivery that include artificial intelligence and machine learning-based applications. The growth and subsequent widespread adoption of such technologies, especially in the field of mental health, has the benefit of providing instant automated access to interventions, improving therapeutical conditions, and reducing the distance gaps between the patient and the clinician. Additionally, it allows patients to receive personalized clinical consultations without the burden of face-to-face interactions.

The scope of this Research Topic is to investigate the impact of telehealth, assistive technologies, and Artificial Intelligence-based applications in the diagnosis, treatment, and monitoring of NDDs. In particular, the Research Topic focuses on the benefits that the adoption of these technologies can bring to the management of mental health, to the outcome of therapeutic interventions, and patients' health in general.

The present Research Topic covers original research articles and case studies on novel technological solutions targeted at patients with neurodevelopmental disorders at early or late developmental stages.

In the work by García et al., the behavioral intention and attitude of mental health practitioners in the potential adoption of virtual humans to provide affect recognition training was explored. Affect recognition, especially in social contexts, is a crucial cognitive skill that allows us to interpret social situations. This skill is impaired in various psychiatric disorders, including schizophrenia. The study stems from the need to provide standardized technology-based therapies for such patients and to evaluate the interest in these techniques from the professionals involved in the field. After viewing a video presentation of the therapist-controlled software, practitioners all showed a positive intention to adopt virtual reality tools for their training, with younger professionals expressing less concern about the knowledge required to use the VR tool and older professionals reporting more potential enjoyment.

Social recognition impairment is not the only NDD symptom lacking standardized therapies or evaluation methods. Atypical motor skills, in fact, are by consensus one of the core and earliest markers in autism spectrum disorder (ASD), regardless of its severity. Minissi et al. administered three virtual reality tasks to both ASD and typically developed (TD) children. These tasks required them to execute precise and goal-directed movements. The authors found that, in the context of the flower task that required participants to move accurately one of their upper arms horizontally, ASD children showed evident motor abnormalities. Specifically, these children displayed notable displacements in the head, body, and upper limbs, along with variations in velocity, acceleration, and deceleration in the limbs or hand. Their execution time was also longer in comparison with TD children.

In another study on the topic of motor abnormalities in ASD children, Milano et al. investigated finer movements of the fingers using motion detection software connected to a tablet. The technology-enhanced setting reproduced the structure of a traditional cognitive battery (the Leiter-3 test) in which children were asked to drag on the screen a series of moving cards near designed placeholders. Hypothesizing the presence of latent features in the movement patterns, the authors analyzed the trajectories using a specific type of Artificial neural network, the variational autoencoder, finding distinctive clusters. The motor features that contributed the most in discriminating the movement pattern of ASD children from TD children were the standard deviation of the speed, the maximum and minimum acceleration, and the standard deviation of the acceleration.

Developmental coordination disorder is a form of motor disability that greatly affects patients' daily lives. Occupational therapy, defined as an intervention aimed at facilitating children in performing daily activities including educational and leisure activities, can greatly improve patients' quality of life. In this context, Shiozu report a case study of applied therapy using Cognitive Orientation to daily Occupational Performance (CO-OP) for tele-occupational therapy. The intervention was delivered to the patient, an 8-year-old child, fully remote and online, with the use of video conferences and e-mails. The authors showed that all predefined goals for the child were achieved and that the child's measured satisfaction was positive, with a marked improvement in performance in comparison with that reported before the intervention.

Bertacchini et al. summarize the AI-enhanced and technology-assisted devices currently in use in the assessment, treatment, and monitoring of NDDs and identify the limitations of such systems along with the open challenges posed by the potential adoption of each of the different tools and techniques available. In the context of social robotics and human-robot interaction, the authors propose an experimental setup for children with ASD based on the humanoid robot Pepper and the more recent large language model ChatGPT. The integrated system, equipped with face-detection, emotion-recognition software, and speech-recognition software, is developed to convey both informal and structured interactions to promote social interactions and deliver cognitive training.

In conclusion, the purpose of the Research Topic is to provide a significant contribution to the comprehension of how these innovative approaches affect the management and treatment of NDDs. Specifically, it aims to outline the ongoing progression through which technological and AI applications, encompassed by pioneering theoretical perspectives, can potentially establish new benchmarks that either supplant or complement conventional medical and psychological interventions.

## Author contributions

DP: Conceptualization, Data curation, Methodology, Software, Writing—original draft. BL-P: Writing—review & editing. DM: Conceptualization, Supervision, Writing—review & editing.

